# Canagliflozin‐induced renal glutathione distribution mapping in non‐diabetic male rat kidneys

**DOI:** 10.14814/phy2.70320

**Published:** 2025-04-13

**Authors:** Guy Watanabe, Shoichiro Horita, Reika Flora Moriya, Yusuke Masuishi, Shingen Misaka, Shu Taira, Kenju Shimomura, Michio Shimabukuro, Junichiro James Kazama

**Affiliations:** ^1^ Department of Nephrology and Hypertension Fukushima Medical University School of Medicine Fukushima Japan; ^2^ Department of Bioregulation and Pharmacological Medicine Fukushima Medical University School of Medicine Fukushima Japan; ^3^ Department of Diabetes, Endocrinology, and Metabolism Fukushima Medical University School of Medicine Fukushima Japan; ^4^ Department of Hygiene and Preventive Medicine Fukushima Medical University School of Medicine Fukushima Japan; ^5^ Faculty of Food and Agricultural Sciences Fukushima University Fukushima Japan

**Keywords:** canagliflozin, glutathione, mass spectroscopy imaging, oxidative stress, SGLT2 inhibitor

## Abstract

Canagliflozin, a sodium glucose cotransporter 2 (SGLT2) inhibitor, has direct renoprotective effects beyond lowering blood glucose levels. The inhibition of sodium reabsorption via SGLT2 reduces the overload on proximal tubules, thereby suppressing the generation of reactive oxygen species (ROS) and preventing a decline in renal function. To clarify the pharmacological mechanism of SGLT2 inhibitor, we investigated the effects of canagliflozin on oxidative stress in the kidneys of normal, non‐diabetic Sprague–Dawley rats. Screening using mass spectrometry images revealed a significant elevation map of the reduced form of glutathione in the renal cortex of canagliflozin‐treated non‐diabetic rats. These results suggest that canagliflozin reduces oxidative stress through ROS scavenging mechanisms. Considering that ROS play major roles in renal dysfunction regardless of diabetes mellitus, these findings suggest that canagliflozin is applicable to a broader range of renal diseases beyond diabetes.

## Graphical abstract

**Canagliflozin and Renal Glutathione:** Mapping its distribution in non‐diabetic male rat kidneys reveals the impact of this SGLT2 inhibitor.

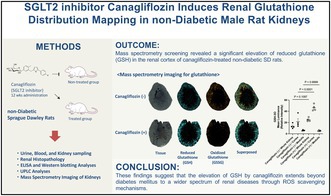

## 1 INTRODUCTION

Recent breakthrough studies on the pharmacological effects of sodium glucose cotransporter 2 (SGLT2) inhibitors, originally developed as diabetes medications, have highlighted their efficacy in treating not only diabetes mellitus but also heart failure and chronic kidney disease (CKD) (Scheen, 2020). Focusing on the direct renal protective effects of SGLT2 inhibitors, the initial evidence came from the EMPA‐REG OUTCOME trial. This trial showed a lower percentage of acute renal failure (including acute kidney injury) in the empagliflozin group compared to the placebo group (Zinman et al., 2015). The CREDENCE trial, which set kidney failure risk as the primary endpoint, demonstrated the effectiveness of canagliflozin (Perkovic et al., 2019). The DAPA‐CKD trial showed that dapagliflozin reduced the risk of a sustained decline in estimated glomerular filtration rate (eGFR) regardless of diabetes (Heerspink et al., 2020). The EMPA‐KIDNEY trial revealed that empagliflozin slowed eGFR decline even in patients with normal or trace albuminuria, suggesting its effectiveness in non‐diabetic CKD patients who are albuminuria‐negative (The EMPA‐KIDNEY Collaborative Group, 2023). These clinical data suggest that SGLT2 inhibitors have direct renoprotective effects beyond lowering blood glucose levels, and the detailed pharmacological mechanisms are being investigated.

Oxidative stress is exacerbated by mitochondrial and endothelial dysfunction, and aberrant activities of reactive oxygen species (ROS)‐producing enzymes including NADPH oxidase, endothelial nitric oxide synthase, xanthine oxidase, and myeloperoxidase under conditions of limited antioxidants (Jomova et al., 2023). This imbalance has been reported to be associated with homocysteine accumulation (Tyagi et al., 2005) and reduced glutathione deficiency, resulting in elevated oxidative stress markers such as 8‐iso‐prostaglandin F_2α_ (8‐iso‐PGF_2α_) (Montuschi et al., 2004), malondialdehyde (Nielsen et al., 1997) and asymmetric dimethylarginine (Tyagi et al., 2005). In CKD patients, impaired superoxide dismutase function (Tbahriti et al., 2013) and glutathione synthesis (Girelli et al., 1993) have been documented. Indoxyl sulfate, a uremic toxin, further exacerbates oxidative stress by promoting NADPH oxidase activity (Dou et al., 2007). These dysregulated enzymes contribute to the oxidative stress environment in CKD patients. The renoprotective effects of SGLT2 inhibitors in hyperglycemia are well‐established, but their impact on the overall oxidative stress environment in non‐diabetic kidneys is not yet fully understood. In the present study, we administered canagliflozin orally to non‐diabetic Sprague–Dawley (SD) rats at a dose of 2 mg/kg per day for 12 weeks to investigate changes in oxidative stress and clarified the pharmacological mechanism of SGLT2 inhibitors. The present study successfully demonstrated direct evidence of improved oxidative stress response in non‐diabetic rats treated with canagliflozin.

## 2 METHODS

### 2.1 Experimental normal SD rats

#### 2.1.1 Experiment 1: Canagliflozin treatment for 12 weeks

All experiments described later were performed in accordance with the relevant guidelines and regulations, including the Animal Research: Reporting of In Vivo Experiments guidelines. SD rats (9–11 weeks old, male) were purchased from CLEA Japan, Inc. (Tokyo, Japan) and housed individually with free access to drinking water in an animal room maintained at 21.5°C with 40%–50% humidity and a 12‐h light–dark cycle. They were subsequently divided into two groups: the canagliflozin‐treated group (*n* = 7) and the non‐treated group (*n* = 8). Canagliflozin was provided by Mitsubishi Tanabe Pharma Corporation (Osaka, Japan). The canagliflozin‐treated group received oral administration of canagliflozin (2 mg/kg/day) suspended in approximately 250 μL of drinking water every morning (7:00–9:00 a.m.) from the age of 14–25 weeks. The rats in both groups were fed a commercial unsterilized rodent diet CE‐2 (CLEA Japan). Non‐fasting blood samples were collected from the tail vein every week from 13 to 25 weeks of age, after which body weight and glucose levels were examined using a glucose test kit with an upper limit of 600 mg/dL (Glutest Neo alpha and Glutest Neo Sensor, Sanwa Kagaku Kenkyusho Co. Ltd., Aichi, Japan).

#### 2.1.2 Experiment 2: Low dose canagliflozin, metformin, 1,3‐butandiol treatment for 2 weeks with unilateral nephrectomy

SD rats (8 weeks old, male) were purchased, and unilateral left nephrectomy was performed at the age of 9 weeks. They were subsequently divided into four groups: the vehicle‐treated group (*n* = 4), the low‐dose canagliflozin‐treated group (*n* = 4), the metformin‐treated group (*n* = 4) and the 1,3‐butandiol‐treated group (*n* = 4). The low‐dose canagliflozin‐treated group and the metformin‐treated group received oral administration of canagliflozin (0.2 mg/kg/day) and metformin (200 mg/kg/day), respectively, dissolved in approximately 250 μL of drinking water every morning (7:00–9:00 a.m.) from 10 to 11 weeks of age. The 1,3‐butandiol‐treated group was fed a diet containing 20% (w/w) 1,3‐butandiol by mixing it with a commercial unsterilized rodent diet CE‐2.

### 2.2 Urine and blood sampling

The rats were each individually housed in metabolic cages (CL‐0353, CLEA Japan Inc.) from 13 to 25 weeks of age, with free access to a commercial unsterilized rodent diet CE‐2 and tap water. Urinary volume, food intake, and water intake were measured weekly. The collected urinary samples were centrifuged at 1000 ***g*** for 3 min, and the supernatants were used for measuring sodium concentrations with LAQUAtwin‐Na‐11 (HORIBA Ltd., Kyoto, Japan) and urinary Cr concentrations via enzymatic methods (SRL Inc., Tokyo, Japan). The remaining urine samples were aliquoted and frozen at −80°C. After their urine samples were collected, the rats were returned to their home cages. Subsequently, blood samples of approximately 500 μL were collected with EDTA (2.5 nmol per tube), then centrifuged at 1710 ***g*** (3000 rpm) for 10 min, and the supernatant was used for sodium concentration measurement using LAQUAtwin‐Na‐11 (HORIBA Ltd.). The remaining supernatant was aliquoted and stored frozen at −80°C, with a portion used for plasma Cr concentration measurement by enzymatic methods (SRL Inc.) to calculate fractional sodium excretion (FE_Na_) and fractional urea nitrogen excretion (FE_UN_).

### 2.3 Euthanasia and kidney sampling

All SD rats, both those without nephrectomy (Experiment 1) and those with left unilateral nephrectomy (Experiment 2), were euthanized at 26 weeks and 12 weeks of age, respectively, 2 h after being transferred to autopsy rooms, via injection of a mixture of anesthetic agents with a 5‐mL/kg injection of an anesthetic mixture (medetomidine [0.0075%, Domitor, Nippon Zenyaku Kogyo Co., Ltd., Fukushima, Japan], midazolam [0.04%, Dormicum, Astellas Pharma Inc., Tokyo, Japan], and butorphanol tartrate [0.05%, Vetorphale, Meiji Seika Pharma Co., Ltd., Tokyo, Japan]), and approximately 10‐mL blood samples were collected into 15‐mL Falcon tubes with EDTA (50 nmol) from the descending vena cava. The samples were then centrifuged at 1710 ***g*** (3000 rpm) for 10 min, and the supernatant plasma was aliquoted and frozen at −80°C in the same way as the weekly collected blood samples. After deep anesthesia was achieved by injecting the three types of anesthetic agents, the rats were perfused intracardially with 0.9% (*w/v*) NaCl solution containing heparin (20 IU/mL). After 15 min of perfusion, the right kidney, both in Experiment 1 and Experiment 2, was extracted from each rat, washed three times with PBS buffer, and the kidney samples were then embedded in super cryo embedding medium (SCEM, Leica Biosystems, Wetzlar, Germany) and frozen in liquid nitrogen for mass spectrometry imaging (MSI). Subsequently, the cortex of the kidney was partially cut out using an optical microscope and microtome blades for Western blotting analyses. In Experiment 1, the rats were next perfused intracardially with saline containing heparin (20 IU/mL), paraformaldehyde, and 0.2% picric acid for 15 min for histological analyses. After perfusion, the remaining kidneys were removed and then cut in the coronal plane, and the pieces were embedded in paraffin before being used for periodic acid‐Schiff (PAS) staining.

### 2.4 Measurement of oxidative stress markers

#### 2.4.1 8‐iso‐PGF_2α_ levels

Renal cortical 8‐iso‐PGF_2α_, a marker of lipid peroxidation, was measured using an enzyme‐linked immunosorbent assay (ELISA) kit (Catalog# ADI‐900‐091, Enzo Life Sciences, NY, USA) according to the manufacturer's instructions. Briefly, renal cortical tissue samples were freeze‐dried using a lyophilizer (EYELA FDS‐1000; Tokyo Rikakikai Co., Ltd., Tokyo, Japan), yielding dried samples ranging from 0.3 mg to 1.9 mg. The dried samples were then dissolved in 1 mL of 2 N NaOH, incubated at 45°C for 2 h, and neutralized with an equal volume of 2 N HCl (1 mL). After centrifugation at 3000 rpm for 10 min, the supernatant was used according to the manufacturer's protocol. Optical density was measured at 405 nm using a Varioskan LUX multimode microplate reader (Catalog# VL0L00D0, Thermo Fisher Scientific, CA, USA).

#### 2.4.2 8‐OHdG levels

Urinary 8‐hydroxy‐2′‐deoxyguanosine (8‐OHdG), a major marker of DNA oxidation, was measured using an ELISA kit (KOG‐200S; Japan Institute for the Control of Aging, Nikken Seil Co., Ltd., Shizuoka, Japan).

#### 2.4.3 Glutathione levels

Renal cortical reduced and oxidized glutathione (GSH and GSSG) levels, indicators of antioxidant status, were quantitatively measured using MALDI‐MSI. Briefly, after perfusion treatment, renal cortical tissue samples were flash‐frozen in liquid nitrogen after being coated with a super cryo‐embedding medium. MALDI‐MSI analyses were then performed as described below.

### 2.5 Preparation of kidney cross‐sections for MSI

After flash‐freezing the embedded kidney samples, the specimen blocks were cut into 10‐μm sections using a cryostat (NX70, Thermo Fisher Scientific) set at −25°C for the chamber and at −20°C for the object holder. The sections were gently mounted on slides coated with indium tin oxide (Bruker Daltonics, MA, USA). Optical images of the sections were obtained using a scanner (GT‐X830, Epson, Tokyo, Japan) before analysis by MALDI‐MSI.

### 2.6 MALDI‐MSI

For ionization of GSH and GSSG, a 40 mg of matrix 9‐aminoacridine (9AA; #92817; Sigma‐Aldrich, MO, USA) was suspended in 6 mL of 70%(*v/v*) ethanol and was sprayed on kidney tissue sections on ITO‐coated glass slides using an automated pneumatic sprayer (TM‐Sprayer, HTX Technologies, NC, USA). For ionization of catecholamines, kidney sections were applied with a 2,4,6‐trimethylpyrylium tetrafluoroborate (Py‐I) solution using an artistic airbrush (Procon Boy FWA Platinum 0.2‐mm caliber airbrush, GSI Creos, Tokyo, Japan) and were subsequently incubated at 60°C for 10 min to generate Py‐I‐labeled catecholamines on the kidney sections. A 10 mg/mL solution of the matrix α‐cyano‐4‐hydroxycinnamic acid (CHCA, Nacalai Tesque Inc., Kyoto, Japan) was suspended in 6 mL of acetonitrile/water/trifluoroacetic acid (50/49/1 *v/v*) and the suspension was sprayed over the slide‐mounted kidney tissue sections using an automated pneumatic sprayer (TM‐Sprayer, HTX Tech., NC, USA). Ionization and imaging of the GSH and GSSG and catecholamines were confirmed using a MALDI−TOF−MS (rapifleX, Bruker Daltonics). For MSI, the laser spot areas were detected by scanning the sections. The laser spot areas (200 shots) were detected with a spot‐to‐spot center distance of 100 μm in each direction of the kidney tissue. Signals between m/z (mass‐to‐charge ratio) 100–800 were corrected. The section surface was irradiated with yttrium aluminum garnet laser shots in the negative ion detection mode for GSH and GSSG and in the positive ion detection mode for catecholamines. The obtained mass spectrometry spectra were reconstructed to mass spectrometry images with a mass bin width of m/z ± 0.05 from the exact mass using FlexImaging 4.0 software (Bruker Daltonics). The peak intensity values of the spectra were normalized by dividing these by the total ion current for semi‐quantitative analysis of GSH, GSSG, and catecholamine distribution in the kidney tissues of control and canagliflozin‐treated rats.

### 2.7 PAS staining

Paraffin‐embedded kidney sections (5‐μm thickness) were deparaffinized with xylene and stained with PAS. Glomerulus images were acquired using an optical microscope (Olympus, Tokyo, Japan).

### 2.8 Western blotting

The renal cortex from 26‐week‐old specimens was homogenized in RIPA buffer (50 mM Tris–HCl pH 7.4; 150 mM NaCl; 1 mM EDTA; 0.1% SDS; 1% Triton X‐100) using sterile homogenizers (Catalog# 9790A, Takara Bio Inc., Shiga, Japan). The homogenate was centrifuged at 15,000 ***g*** for 10 min, and 15 μL of the supernatant was collected. The samples were then mixed with 5 μL of lithium dodecyl sulfate loading buffer containing 100 mM dithiothreitol and boiled at 95°C for 10 min. The boiled samples were loaded onto 4%–12% Bis‐Tris NuPAGE gels (Catalog# NP0322, Invitrogen, Paisley, UK) and electrophoretically transferred to a PVDF membrane. This membrane was blocked with PBS buffer containing 2% (w/v) BSA and 0.1% Triton X‐100, then incubated with primary antibodies (anti‐Nrf2 [1:10000, Catalog# 16396‐1‐AP, Proteintech, IL, USA] or anti‐GPx4 [1:2000, Catalog# 67763–1‐1G, Proteintech], and anti‐GAPDH [1:20000, Catalog# 10494‐1‐AP, Proteintech]) for 1.5 h, followed by incubation with the secondary antibody (1:20000, Catalog# RGAR001, HRP‐conjugated goat anti‐rabbit IgG, Proteintech) for 1 h. The blots were developed using chemiluminescence with luminol reagent (Clarity Western ECL Substrate, Catalog# 1705060, Bio‐Rad Laboratories, CA, USA), and the bands were visualized using ChemiDoc XRS+ gel imaging system (Bio‐Rad Laboratories).

### 2.9 LC–MS/MS

LC–MS/MS analysis was performed on 10 μL of each urine sample using a Vanquish UHPLC system coupled with Orbitrap Exploris 240 (Thermo Fisher Scientific, Germany). An Acclaim 120 C18 column (100 mm × 2.1 mm, 3 μm; Thermo Fisher Scientific Inc.) was used for separation at a flow rate of 300 μL/min. Solvent A was 0.1% (*v/v*) FA in water, and solvent B was 0.1% FA (v/v) in ACN. The samples were gradually separated using a gradient of 1%–30% solvent B over 5 min. The samples were ionized by heated electrospray ionization in positive mode, and DA was detected by parallel reaction monitoring (PRM) scanning at a normalized collision energy of 30 and a mass resolution of 60,000. The scan range and the maximum injection time were set to automatic mode. The PRM scans were triggered by the DA precursor ion at m/z 154.086. PRM data were processed using the Xcalibur software package version 4.7 (Thermo Fisher Scientific Inc.). Xcalibur integrates the DA fragment ion at m/z 137.0596, then uses linear regression to calculate the concentration from the standard curve.

### 2.10 UPLC

Concentrations of DA in plasma and urine were determined by hydrophilic interaction chromatography using an ultra‐performance liquid chromatography (UPLC) system (Waters Corp., Milford, MA, USA) with fluorometric detection. Prior to injection into the UPLC system, DA was extracted using a monolithic solid‐phase extraction column (MonoSpin PBA; GL Sciences, Tokyo, Japan) according to the manufacturer's instructions with minor modifications. In brief, plasma samples were centrifuged at 10,000 ***g*** for 5 min, and the supernatants were mixed with 1.5 M HEPES buffer (pH 8.5) (Nacalai Tesque Inc.) at a ratio of 9:1. Urine samples were diluted 1:1 with 50 mM ammonium acetate buffer (pH 6.0) (FUJIFILM Wako Pure Chemical Corporation, Osaka, Japan) and passed through an Evolute AX Express cartridge (30 μm, Biotage, Uppsala, Sweden) on a vacuum manifold to remove anionic contaminants in urine. The eluates were mixed with 1.5 M HEPES buffer (9:1). Next, the plasma and urine samples were applied to MonoSpin PBA columns that were preconditioned with 1% acetic acid (FUJIFILM Wako Pure Chemical Industries) and 0.1 M HEPES buffer (pH 8.5). The samples were washed with 0.1 M HEPES buffer (pH 8.5) and eluted with 1% formic acid/acetonitrile (70:30, v/v) (FUJIFILM Wako Pure Chemical Industries). After 5‐fold dilution with acetonitrile and subsequent filtration through a 0.22‐μm filter membrane (Millex‐LG, Millipore, MA, USA), 5 μL of the sample was injected into the UPLC system. Chromatographic separation was performed using an ACQUITY UPLC BEH Amide column (particle size 1.7 μm; 2.1 mm × 50 mm, Waters) at 40°C with a flow rate of 0.6 mL/min. The mobile phase consisted of 20 mM ammonium formate (FUJIFILM Wako Pure Chemical Industries) with 0.2% formic acid and 0.1 mM EDTA (Dojindo, Kumamoto, Japan) (A) and acetonitrile (B). The linear gradient was as follows: 0–2.0 min, 93% B; 2.0–9.0 min, 93%–85% B; and 9.0–13.0 min, 93% B. DA was detected at an excitation wavelength of 295 nm and an emission wavelength of 345 nm. The limit of quantification for DA was 10 nM.

### 2.11 Urinary calculation

The urinary levels of DA were determined by UPLC analyses as described in the previous section. Plasma and urinary Cr levels, as well as plasma albumin levels, were measured using enzymatic methods (SRL, Inc.). The values of creatinine clearance (CCr) were calculated using the following equation:

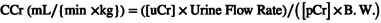

where Urine Flow Rate is the amount of excreted urine collected per minute, B.W. is body weight (kg), and uCr and pCr are urinary Cr and plasma Cr, respectively.

The values of a series of excretion rates (FE_UN_, FE_Na_) were calculated using the following equations:

$${\mathrm{FE}}_{\mathrm{UN}}\;\left(\%\right)=\left\{\left(\left[\mathrm{uUN}\right]/\left[\mathrm{pUN}\right]\right)/\left(\left[\mathrm{uCr}\right]/\left[\mathrm{pCr}\right]\right)\right\}\times 100$$

$${\mathrm{FE}}_{\mathrm{Na}}\;\left(\%\right)=\left\{\left(\left[\mathrm{uNa}\right]/\left[\mathrm{pNa}\right]\right)/\left(\left[\mathrm{uCr}\right]/\left[\mathrm{pCr}\right]\right)\right\}\times 100$$

### 2.12 GPx activity

Glutathione peroxidase (GPx) activity in the renal cortex was measured colorimetrically using a procedure for the Glutathione Peroxidase Activity Assay Kit (LS‐K669‐100; LifeSpan BioSciences, Inc., WA, USA). Kidney samples were collected in microcentrifuge tubes and prepared with radio‐immunoprecipitation assay buffer without SDS (purchased from Nacalai Tesque Inc.) as a stock. These samples were homogenized until they had a uniform consistency and were then flash‐frozen at −80°C to achieve a concentration of 100 μg/μL. To prepare for the measurements, the following reagents were mixed and incubated in each well of a 96‐well plate: 10 μL of 10 μM β‐NADPH (Oriental Yeast Co., Ltd., Tokyo, Japan), 6.08 μg of GSH (FUJIFILM Wako Pure Chemicals Co., Ltd), 0.1 U of glutathione reductase (Oriental Yeast Co., Ltd.), and 10 μL of cumene hydroperoxide (#C2223, Tokyo Chemical Industry, Co., Ltd., Tokyo, Japan) dissolved in 5% ethanol. The appropriately diluted kidney samples above were then incubated, and the reduction of A_340_ nm, indicating the degradation of β‐NADPH, was measured every 30 s using a spectrophotometer. The reduction rate per minute was calculated and used as the measure of GPx activity.

### 2.13 Statistical analysis

Continuous data measured every week were presented as the means ± standard deviations (SD), and statistical analyses were performed using an unpaired *t*‐test between the control and canagliflozin‐treated groups for each week of the study. Single‐measurement data were presented as the means ± standard error of the mean (SEM), and statistical analyses were performed using an unpaired *t*‐test between the control and canagliflozin‐treated groups. Luminance intensity from mass spectrometry imaging was analyzed using ImageJ software version 1.51 (National Institutes of Health, MD, USA); the intensity was estimated in triplicate, and the resulting data were analyzed statistically using one‐way ANOVA followed by Tukey's multiple‐comparison test, using data from all tissues (whole kidney, cortex, medulla), comparing the mean of each with the mean of every other. All statistical analyses were performed using GraphPad Prism 10.2.3 (GraphPad Software, Inc., CA, USA) for MacOS, version 10.14.6. In the manuscript, *p* values of <0.01 were considered statistically significant.

## 3 RESULTS

### 3.1 Long‐term canagliflozin treatment on non‐diabetic Sprague–Dawley rats

**FIGURE 1:**
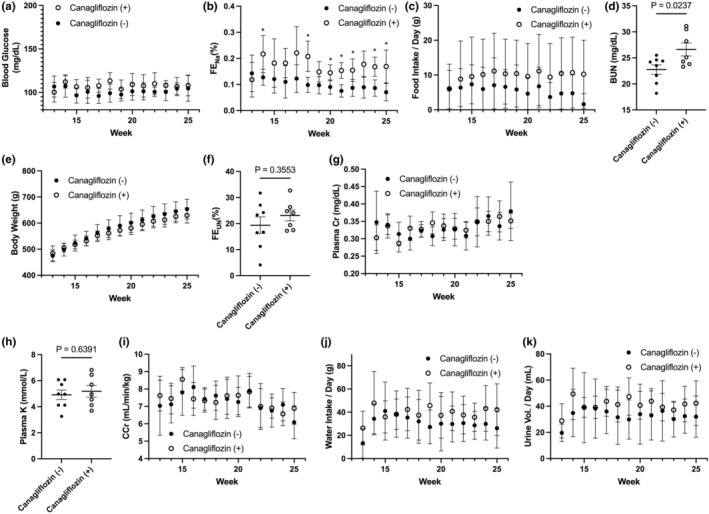
Data from Experiment 1. (a–c, e, g, i–k) Mean values (± SD) of weekly measurements from 13 to 25 weeks of age in non‐treated rats (*n* = 8) and canagliflozin‐treated rats (*n* = 7; treatment started at 14 weeks of age): Blood glucose (a); fractional sodium excretion (FE_Na_) (b); food intake per day (c); body weight (e); plasma creatinine (g); creatinine clearance (i); water intake per day (j); and urinary volume per day (k). Asterisks (*) indicate statistical significance at *p* < 0.01 (unpaired *t*‐test). (d, f, h) Mean values (± SEM) of blood urea nitrogen (d); fractional urea nitrogen excretion (FE_UN_) (f); plasma potassium concentration (h) at 26 weeks for the non‐treated rats (*n* = 8) and canagliflozin‐treated rats (*n* = 7). Exact *p*‐values are shown above each figure (unpaired *t*‐test).

To investigate the long‐term renal protective effects of canagliflozin, we conducted a 12‐week daily administration trial of canagliflozin in rats from age 14–25 weeks in Experiment 1. As a result, regardless of whether canagliflozin treatment was administered or not, the blood glucose level consistently remained within the normal range of 96.0–114.4 mg/dL (Figure 1a). Canagliflozin‐treated SD rats exhibited an increase in urinary glucose excretion and FE_Na_ (Figure 1b), and these rats also showed a trend toward increased food consumption under free‐feeding conditions (Figure 1c), which possibly led to a slight increase in blood urea nitrogen (BUN) (Figure 1d) and helped minimize weight loss, leading to weight maintenance (Figure 1e). Considering there is no difference in FE_UN_ (Figure 1f), the increased BUN does not reflect a decline in renal function but rather a hypercatabolic state.

The average weight of the non‐treated rats increased from 474.8 ± 22.6 g at 13 weeks of age to 653.5 ± 37.9 g at 25 weeks, representing a 37.6% increase. In contrast, the average weight of the canagliflozin‐treated rats increased from 483.9 ± 28.6 g at 10 weeks to 629.4 ± 29.4 g at 25 weeks, showing a 30.1% increase. The *p* value measured every week showed a decreasing trend (from 0.50 at 13 weeks to 0.20 at 25 weeks), suggesting that sustained canagliflozin treatment could potentially mitigate body weight gain. However, it should be noted that the results obtained in this study do not reflect the potential effects of canagliflozin on body weight reduction.

Notably, considering similar FE_UN_ (Figure 1f), plasma creatinine (Cr) levels (Figure 1g) and plasma potassium levels (Figure 1h), the canagliflozin‐treated SD rats did not exhibit any signs of kidney dysfunction. Furthermore, there were no significant differences in CCr (Figure 1i) or glomerular filtration rate between the canagliflozin‐treated and non‐treated SD rat groups, and the “initial dip”, which is clinically observed during the initial phase of SGLT2 inhibitor treatment in diabetic patients, was not noted in our study. Throughout the entire observation period, the canagliflozin‐treated group showed a slight upward trend in daily water intake (Figure 1j) and urinary volume (Figure 1k) compared to the non‐treated SD group. Furthermore, histopathological findings revealed no significant difference in the average glomerular sizes in both groups. Additionally, tubulointerstitial lesions, lymphocyte infiltration, and structural changes in the tubules were not observed in either group.

### 3.2 Oxidative stress assessment via GSH/GSSG

**FIGURE 2:**
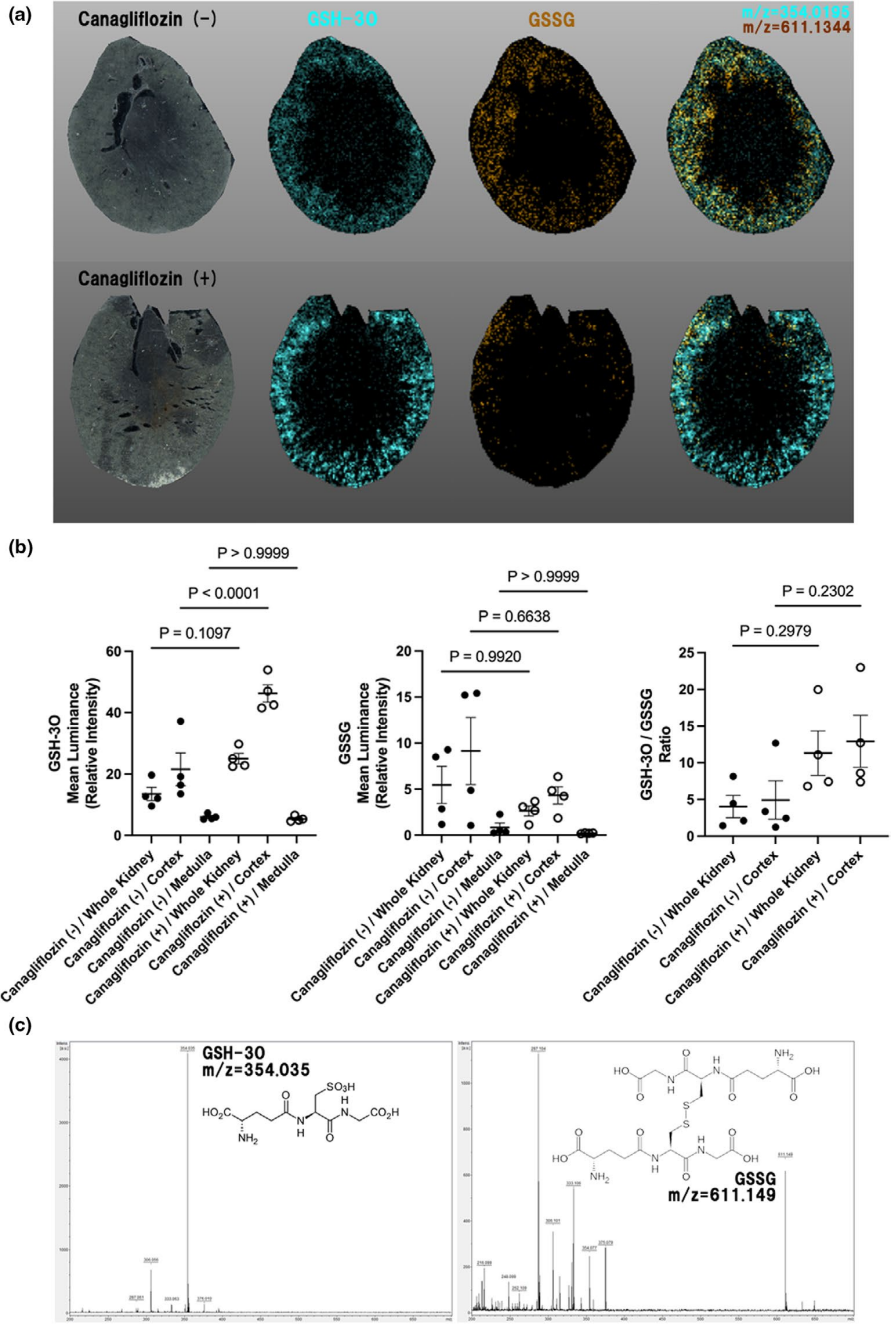
Data from Experiment 1. (a) Superposition of transverse sections of the representative kidneys in the control non‐treated SD rats (upper) and canagliflozin‐treated SD rats (below) at 26 weeks, with GSH‐3O (shown in cyan) and GSSG (shown in orange) localization by mass spectroscopy imaging. (b) Mean values (± SEM) of quantitative intensity of GSH (cyan), GSSG (orange) and GSH/GSSG ratio in the whole kidney, cortex, and medulla calculated from mass spectroscopy imaging. After performing one‐way ANOVA followed by Tukey's multiple‐comparison test, exact *p*‐values are shown only for paired tissues. (c) The dominant peaks of GSH‐3O and GSSG in the GSH and GSSG standard samples, which were subsequently mapped to kidney samples.

Although there were no significant histopathological differences, we could observe a different oxidative stress environment in the kidneys of both the canagliflozin‐treated and non‐treated SD rats in Experiment 1. Antioxidants such as GSH (consisting of three amino acids γ‐Glu‐Cys‐Gly) and GSSG (consisting of two molecules of disulfide‐bonded GSH) are potential indicators of oxidative stress in the kidneys (Asensi et al., 1999). To assess oxidative conditions directly, we performed MSI analyses, successfully visualizing the oxidized and reduced forms of glutathione (Figure 2a,b; Figure S1). The dominant peak of GSSG was found around m/z = 611.149 in a standard sample. Notably, the dominant peak of GSH in standard samples was observed around m/z = 354.035 instead of m/z = 306.056, which is theoretically relevant to GSH (Figure 2c). The observed m/z indicates the addition of three oxygen atoms to GSH, corresponding to Cys‐3O. It is known that the Cys‐3O was detected as a dominant peak when Cys was negatively ionized in the presence of oxygen (Cys‐SH to Cys‐SO_3_H) (Wende et al., 2020). Therefore, the m/z peak of GSH‐3O is considered to represent GSH in the kidneys. The distribution maps of GSH‐3O (m/z = 354.0195) and GSSG (m/z = 611.1344) obtained from MSI reflect the oxidative environment in the kidneys, with the canagliflozin‐treated SD rats showing significantly higher GSH compared to the non‐treated SD rats (Figure 2b). While it has been reported that the quantification of urinary GSH is challenging due to peptide degradation (Mulder & Kostyniak, 1985), these distribution maps clearly visualized the renal GSH status, demonstrating the effects of canagliflozin on renal GSH levels under non‐diabetic conditions.

**FIGURE 3:**
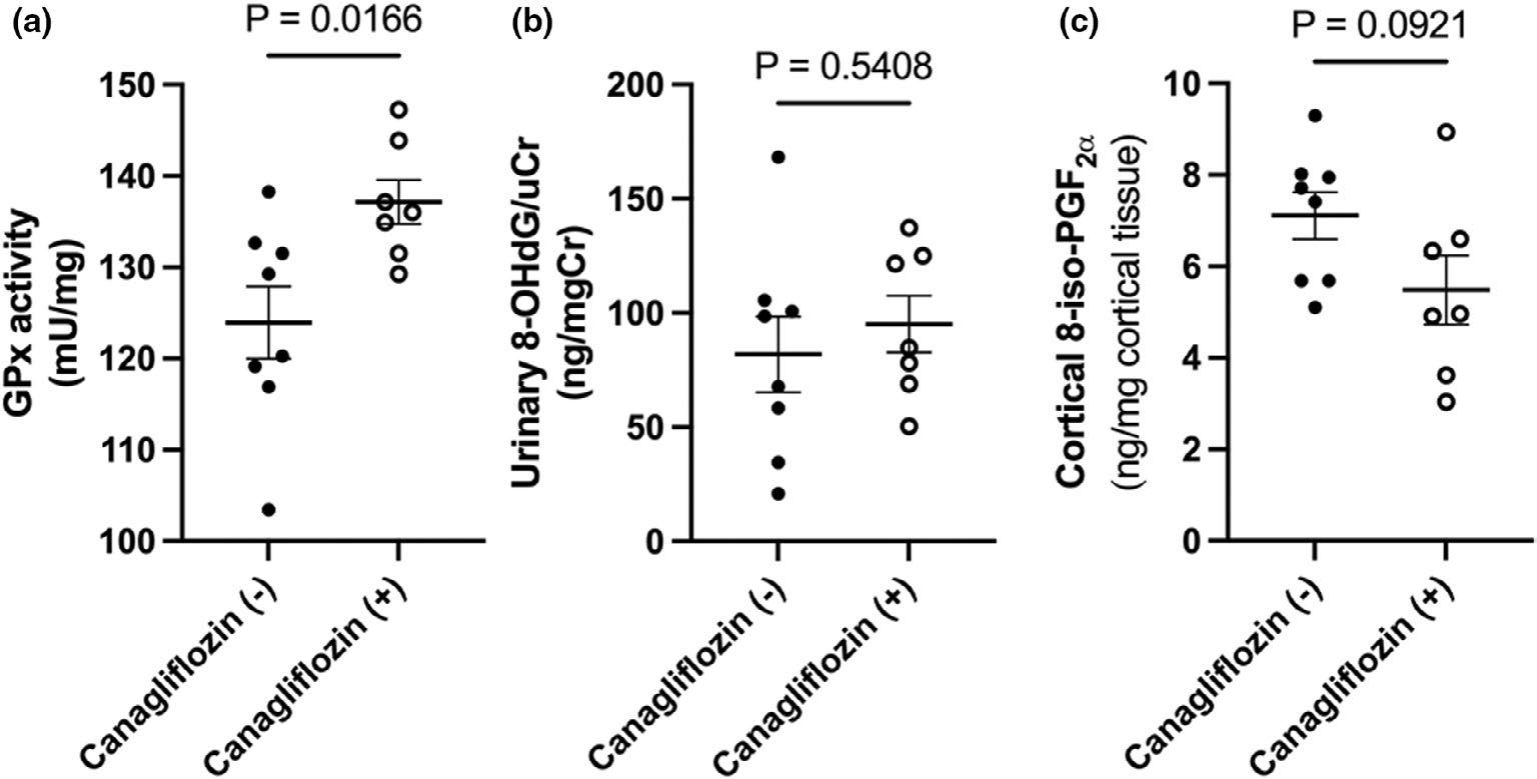
Data from Experiment 1. (a) Mean values (± SEM) of glutathione peroxidase activity determined in homogenized renal cortex samples by measuring the reduction of NADPH absorbance at 340 nm using a spectrophotometer. (b, c) Mean values (± SEM) of representative oxidative stress markers. 8‐OHdG, a marker of oxidative DNA damage, in urine (b), and 8‐iso‐PGF_**2**α_, a marker of lipid peroxidation, in the renal cortex (c). Exact *p*‐values are shown above each figure (unpaired *t*‐test).

Next, to investigate the activity of GPx, we used a calorimetric method to determine its contents. Although Western blot analyses did not show any difference in GPx4 expression (Figure S1), this method revealed a tendency toward increased glutathione reduction, as evidenced by increased overall GPx activity in the canagliflozin‐treated SD rats (Figure 3a). These results indicate that enhanced GPx activity partly contributes to maintaining cortical GSH levels while sustaining the potential antioxidant condition during canagliflozin treatment in non‐diabetic SD rats.

### 3.3 Effects of canagliflozin on urinary 8‐OHdG and renal cortical 8‐iso‐PGF_2α_

To investigate oxidative stress markers beyond GSH, we examined urinary 8‐OHdG, a major form of DNA damage induced by ROS, in both control and canagliflozin‐treated rats in Experiment 1. No significant differences were observed in the levels or synthesis velocity of 8‐OHdG, a well‐established marker of oxidative stress, between the two groups (Figure 3b). Next, to directly assess oxidative stress in renal tissue, cortical 8‐iso‐PGF_2α_ was examined after vacuum freeze‐drying to detect lipid peroxidation. Consistent with the urinary 8‐OHdG results, there was no significant difference in cortical 8‐iso‐PGF_**2**α_ levels between canagliflozin‐treated and non‐treated SD rats (Figure 3c). This suggests that renal cortical tissue oxidative stress was not affected by the increased renal cortical GSH in canagliflozin‐treated SD rats in this study.

### 3.4 Effects of metformin and 1,3‐butanediol on GSH/GSSG

To further investigate the mechanisms behind the observed increase in GSH in Experiment 1, we conducted Experiment 2 to test whether lower doses of canagliflozin, metformin, or 1,3‐butandiol affected cortical GSH levels. To explore the role of mitochondrial function in proximal tubular cells, we administered metformin, a known mitochondrial complex I inhibitor (Albalawy et al., 2024; Omachi et al., 2021). Given previous reports that empagliflozin‐induced increases in ketone bodies suppress renal injury via mTORC1 inhibition (Tomita et al., 2020), we also examined the relationship between this effect and GSH by administering a ketone body precursor 1,3‐butandiol. Additionally, we tested whether a lower dose of canagliflozin (one‐tenth of the dose used in Experiment 1) could also increase GSH levels. Following a 2‐week administration and feeding period, we conducted an MSI analysis of GSH using the remaining kidneys. While a significant difference in reduced GSH was observed with a daily administration of 2 mg/kg/day of canagliflozin for 12 weeks in Experiment 1 (Figure 2a,b), we did not observe any increase in GSH in the groups treated with daily administration of 0.2 mg/kg/day of canagliflozin, 200 mg/kg/day of metformin, and 20%(*w/w*) 1,3‐butandiol for 2 weeks in Experiment 2 (Figure S3). The absence of observed changes suggests that either the interventions were insufficient to elicit measurable increases in GSH levels, or that the observed increase in GSH in Experiment 1 was a secondary phenomenon. Given that the renoprotective effects of metformin require prolonged administration (Omachi et al., 2021), it is plausible that extended treatment durations, as well as dose‐dependent factors, are necessary to achieve an increase in GSH in the renal cortex.

### 3.5 Effects of canagliflozin on dopamine excretion

Our previous study found that physiological levels of peripheral DA are involved in oxidative stress and exert renoprotective effects revealed by decreased urinary 8‐iso‐PGF_**2**α_ and decreased plasma CysC (Horita et al., 2024). Given that both SGLT2 inhibitors and DA suppress sodium(Na^+^)/hydrogen(H^+^) exchange 3 (NHE3) activity in the renal proximal tubule (Hu et al., 2001; Masuda et al., 2018; Onishi et al., 2020; Wiederkehr et al., 2001), we hypothesized a potential pharmacological or physiological link between SGLT2 inhibitor and DA. To explore changes in DA excretion with canagliflozin treatment, we measured the urinary DA excretion for 9 weeks, from weeks 13 to 22 in Experiment 1. Throughout the entire observation period, DA excretion remained unchanged (Figure S4). Consistent with this, our MSI analyses at 26 weeks of age showed no significant differences in L‐dihydroxyphenylalanine (L‐DOPA) levels, a DA precursor (Figure S5). While canagliflozin may not affect NHE3 activity under non‐diabetic conditions (Masuda et al., 2018; Onishi et al., 2020), it is unlikely that the renal dopaminergic system plays a role in the observed increase in GSH following canagliflozin treatment in non‐diabetic conditions.

## 4 DISCUSSION

While chronic hyperglycemia is known to elevate renal oxidative stress in diabetic patients, renal oxidative stress can also be elevated in normoglycemic individuals due to enhanced pro‐oxidant enzyme‐induced ROS production and depleted antioxidants. In this study, we demonstrated that long‐term canagliflozin treatment in non‐diabetic SD rats resulted in an increase in GSH within the cytoplasm or interstitium of the kidneys. Given that angiotensin II or a high salt diet can induce NADPH oxidase activity and mitochondrial ROS production (Gill & Wilcox, 2006; Sachse & Wolf, 2007), and that increased GSH is known to suppress ROS, it is plausible that the observed increase in GSH will contribute to the inhibition of CKD progression.

In the current study, SD rats treated with canagliflozin displayed elevated renal GSH levels and a trend of increased GPx activity when compared to the control SD rats. It is noteworthy that a previous study reported similar findings, where diabetic mice treated with ipragliflozin reduced renal cortical GSSG levels (Tanaka et al., 2018). This consistency with our results suggests that the increase in renal cortical GSH levels is not solely due to canagliflozin, which inhibits both SGLT1 and SGLT2 like sotagliflozin, but could also be attributed to the class effects of SGLT2 inhibitors that specifically inhibit SGLT2, such as dapagliflozin, empagliflozin, and ipragliflozin. It is unlikely that the increased GSH levels were due to additional SGLT1 inhibition, which increases circulating glucagon‐like peptide 1 (Powell et al., 2013, 2017) or the macula densa SGLT1‐NOS1‐tubuloglomerular feedback pathway (Zhang et al., 2019), in the renal cortex by canagliflozin.

Our study has several limitations. First, Experiment 1 was concluded when the rats were 25 weeks of age. It is conceivable that extending the duration of canagliflozin treatment might have resulted in a significant decrease in cortical oxidative stress marker 8‐iso‐PGF_2α_. This potential outcome warrants further investigation. Second, Experiment 1 was conducted using non‐diabetic healthy rats, in which renal function decline is less likely to occur. we hypothesize that using rats with a higher incidence of renal function decline would enhance the detection of antioxidative defenses due to increased GSH. Third, the daily oral administration of canagliflozin inevitably involves animal handling, which can potentially influence the data.

In conclusion, our pharmacological analyses demonstrate that canagliflozin significantly increases GSH levels in the renal cortex of non‐diabetic SD rats. Given that ROS play pivotal roles in renal dysfunction, these findings suggest that the renoprotective effects of canagliflozin may extend beyond diabetes mellitus to a wider spectrum of renal diseases.

## AUTHOR CONTRIBUTIONS

S.H. conceived and designed the project. G.W., S.H., and R.F.M. collected urine and blood samples. G.W., S.H., and R.F.M. performed the dissection of the SD rats and collected blood and kidney samples. G.W., S.H., and S.T. performed MSI experiments and analyses. S.H., Y.M., and S.M. performed the LC–MS/MS and UPLC experiments and the data analyses. S.H. performed the Western blot analyses. G.W. and S.H. performed the pathological examinations. K.S. provided support to facilitate the acquisition of canagliflozin for this research; M.S. and J.J.K. provided valuable insights and comments for finalizing this manuscript. G.W. and S.H. drafted and revised the manuscript.

## FUNDING INFORMATION

This work was partly funded by Grant‐in‐Aid for Scientific Research 24K11414 from the Japan Society for the Promotion of Science (to S.H.).

## CONFLICT OF INTEREST STATEMENT

The study was supported by Mitsubishi Tanabe Pharma Corporation, who provided the canagliflozin. They did not play any role in the data collection, analysis, and interpretation of data, or writing of the manuscript.

## ETHICS STATEMENT

All animal experiments were conducted in accordance with the guidelines of the Animal Research Committee and the Experimental Animal Center of Fukushima Medical University (approval nos. 2022115 and no. 2024037).

## Supporting information


Figure S1.

## Data Availability

Data will be made available upon reasonable request.
