# Supplementary material for: Canagliflozin‐induced renal glutathione distribution mapping in non‐diabetic male rat kidneys
Source: Physiol Rep. 2025 Apr 13;13(7):e70320. doi: 10.14814/phy2.70320 (PMC11994861; doi:10.14814/phy2.70320)

Supplementary Material

Canagliflozin-Induced Renal Glutathione Distribution Mapping in non-Diabetic Male Rat Kidneys

Guy Watanabe[a,b], Shoichiro Horita[c], Reika Flora Moriya[a], Yusuke Masuishi[d], Shingen Misaka[b], Shu Taira[e], Kenju Shimomura[b], Michio Shimabukuro[c], Junichiro James Kazama[a]

[a]*Department of Nephrology and Hypertension, Fukushima Medical University School of Medicine, Fukushima 960-1247, Japan*
[b]*Department of Bioregulation and Pharmacological Medicine, Fukushima Medical University School of Medicine, Fukushima 960-1295, Japan*
[c]*Department of Diabetes, Endocrinology, and Metabolism, Fukushima Medical University School of Medicine, Fukushima 960-1295, Japan*
[d]*Department of Hygiene and Preventive Medicine, Fukushima Medical University School of Medicine*
[e]*Faculty of Food and Agricultural Sciences, Fukushima University, Fukushima 960-1296, Japan*

Supplementary Figure 1. Data from Experiment 1. Transverse sections of the all kidneys in the control non-treated SD rats (upper) and canagliflozin-treated SD rats (below) at 26 weeks, showing tissue sections (A) and the localization of GSH-3O (shown in cyan; B), GSSG (shown in orange; C), and the superposed localization of GSH and GSSG (D) by mass spectroscopy imaging.

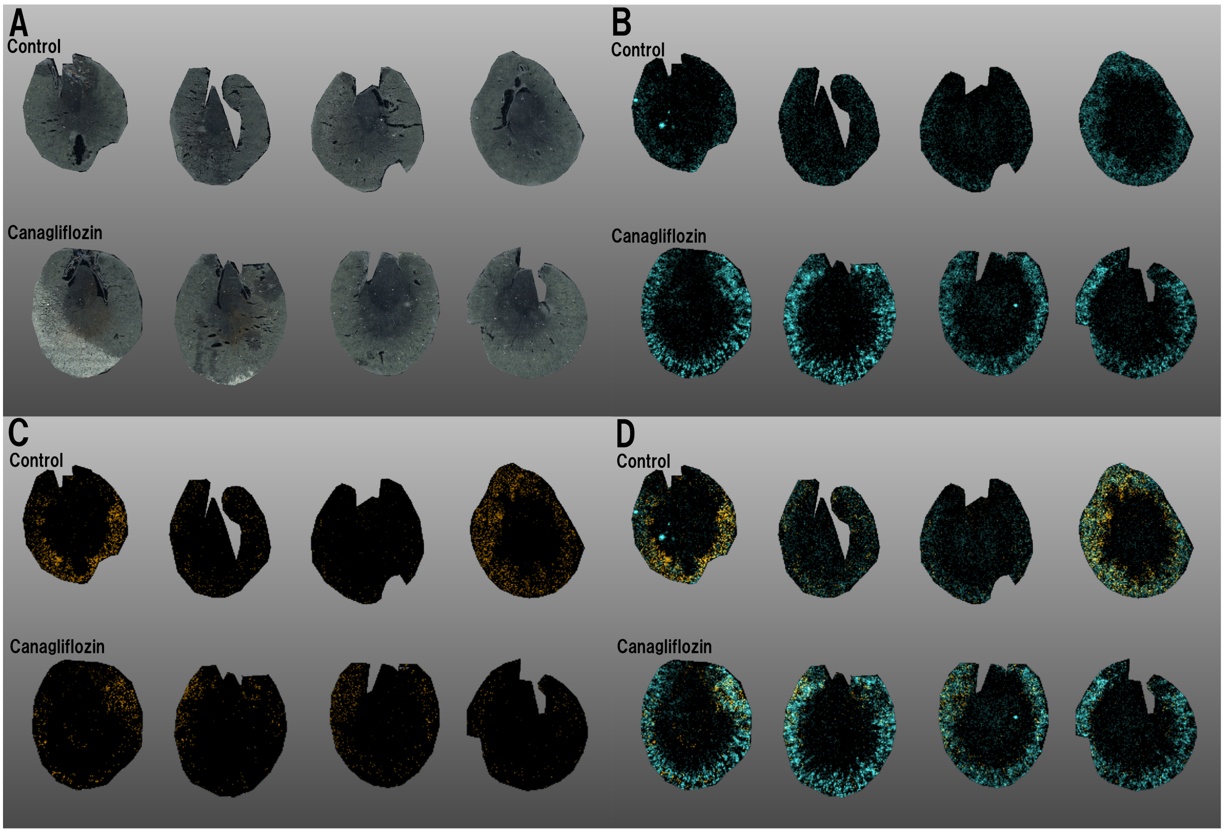

Supplementary Figure 2. Data from Experiment 1. Immunoblotting analyses reveal no detectable GPx4 and Nrf2 expression in both non-treated and canagliflozin-treated SD rats.

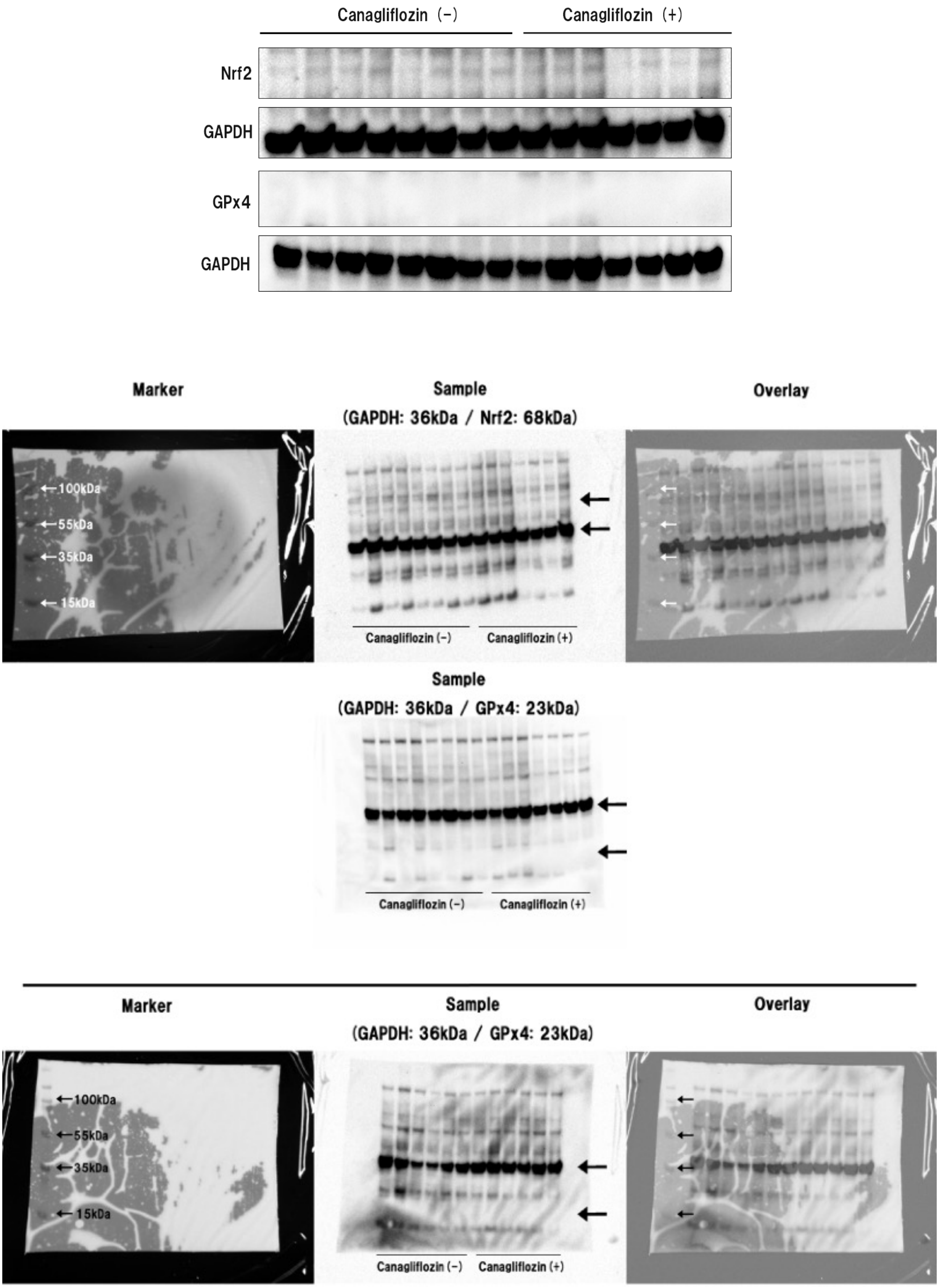

Supplementary Figure 3. Data from Experiment 2. (A–D) Mass spectrometry imaging of GSH-3O (cyan; B) and GSSG (orange; C) localization, and the superposed localization of GSH and GSSG (D) in kidney tissues sections (A) from SD rats. Representative kidney sections are shown for vehicle-treated (upper left; control), low-dose canagliflozin-treated (upper right), metformin-treated (lower left), and 1,3-butandiol-fed (lower right) after a 2-week administration and feeding period. The *m/z* values for GSH-3O and GSSG are 354.058 and 611.140, respectively. (E) Relative intensity of GSH-3O and GSSG (*n* = 4 per group) is normalized to the control group (set to 1.0). After performing one-way ANOVA followed by Tukey's multiple-comparison test using data from all tissues, exact *p*-values are shown only for paired tissues.

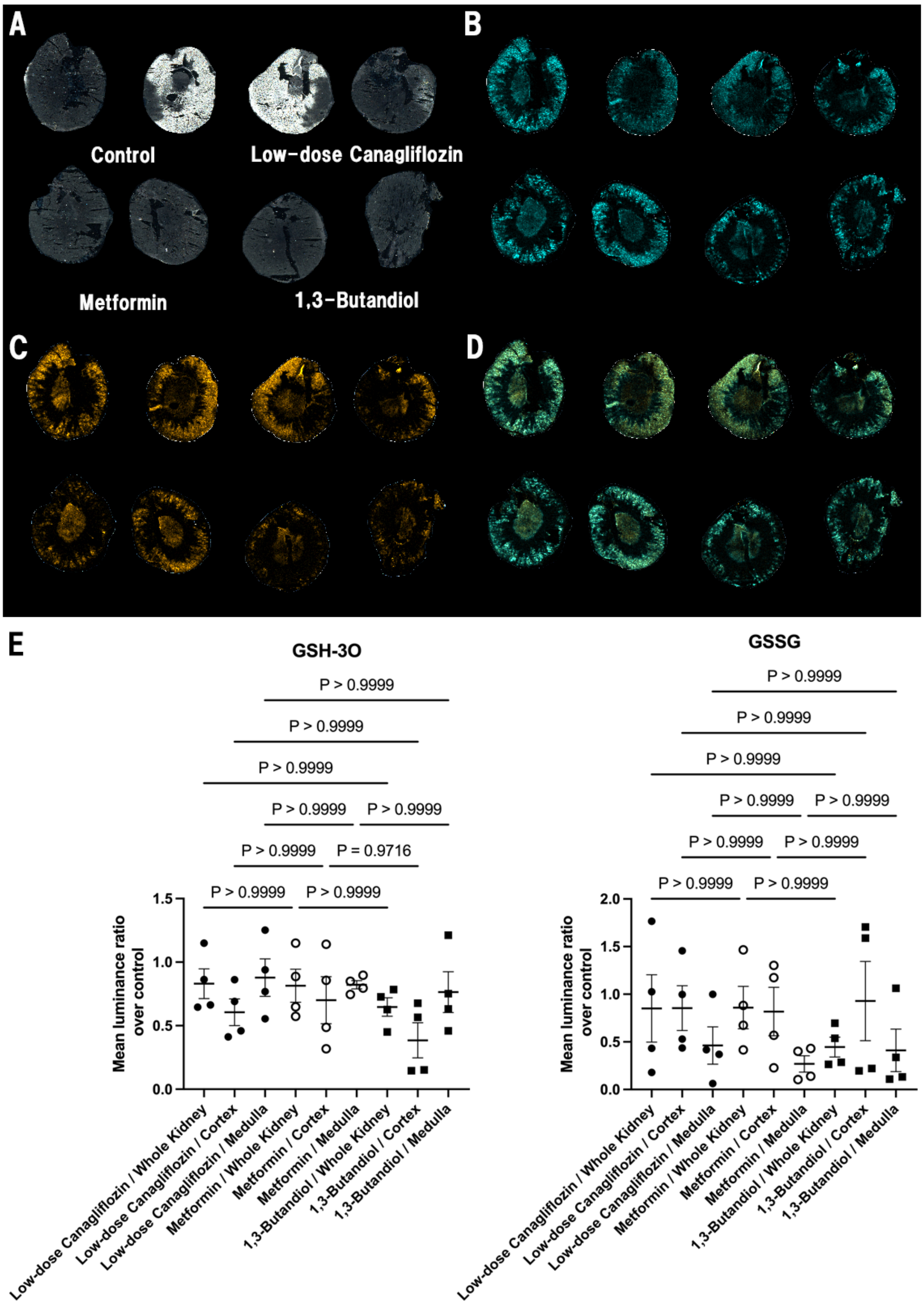

Supplementary Figure 4. Data from Experiment 1. Mean values (± SD) of u-DA/Cr for the non-treated rats (*n* = 4), and canagliflozin-treated rats (*n* = 4; treatment started from 14 weeks of age), measured weekly from the ages of 13 to 22 weeks.

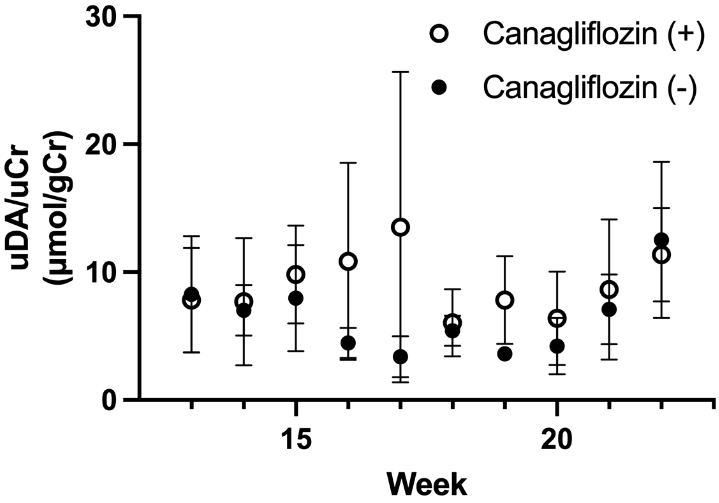

Supplementary Figure 5. Data from Experiment 1. (A) Superposition of transverse sections of the representative kidneys in the control non-treated SD rats (upper) and canagliflozin-treated SD rats (below) at 26 weeks, with DA (shown in red) and L-DOPA (shown in green) localization by mass spectroscopy imaging. (B) Mean values (± SEM) of quantitative intensity of DA (red) and L-DOPA (green) in the whole kidney, cortex, and medulla calculated from mass spectroscopy imaging. After performing one-way ANOVA followed by Tukey's multiple-comparison test, exact *p*-values are shown only for paired tissues.

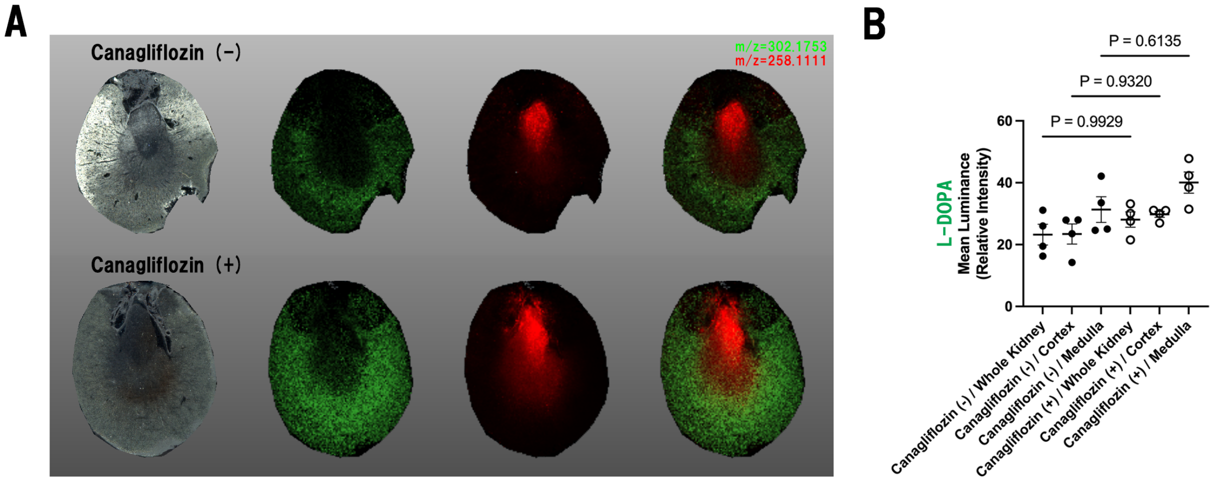

Supplement: Supplementary file 1 — Figure S1. [file PHY2-13-e70320-s001.pdf]
